# Long Term Therapeutic Efficacy of a Soft Monobloc Mandibular Advancement Device in Adults with Obstructive Sleep Apnea

**DOI:** 10.1155/2015/408469

**Published:** 2015-01-06

**Authors:** Fabiana Ballanti, Salvatore Ranieri, Alberto Baldini, Paola Cozza

**Affiliations:** Department of Clinical Science and Translational Medicine, Orthodontics, University of Rome “Tor Vergata”, Viale Oxford 81, 00133 Rome, Italy

## Abstract

*Aim*. To evaluate the long term (48 months) therapeutic efficacy of a soft monobloc mandibular advancement device in adult patients with mild or moderate obstructive sleep apnea. *Methods*. The study population comprised 28 patients (6 female and 22 male, mean age 52.2 ± 6.8 years) affected by obstructive sleep apnea. After a baseline medical and somnographic examination, a functional examination of the stomatognathic system, and a questionnaire focused on sleep-related qualities and a daytime somnolence, each patient received an individual device. Two follow-ups were made 6 months (T1) and 48 months (T2) after soft monobloc mandibular advancement device treatment had been initiated, and all initial examinations were repeated. *Results*. The statistical analysis showed a significant decrease in body mass index value between T1 and T2 (*ρ* = 0,012), an increase of Epworth sleepiness scale value between T1 and T2 (*ρ* = 0,012), and a significant improvement and decrease of apnea/hypopnea index between T0 and T1 (*ρ* = 0,010) and between T0 and T2 (*ρ* = 0,013). *Conclusion*. Treatment with the soft monobloc mandibular advancement device is a therapeutic solution with long term and stable effects (48 months) for patients suffering from mild or moderate obstructive sleep apnea.

## 1. Introduction

Long term efficacy with mandibular advancement devices (MADs) in the treatment of obstructive sleep apnea (OSA) is understudied.

Obstructive sleep apnea (OSA), which affects between 2% and 7% of middle aged adults, is characterized by disruption of normal sleep architecture due to complete or partial obstruction of respiratory airflow. It produces arousal in an attempt to reopen the airways that diminishes the quality of sleep [1–3].

If untreated, the reduction of blood oxygen saturation due to airflow obstruction could determine serious cerebral, bronchopulmonary and cardiovascular complications such as memories disorders, pulmonary hypertension, atherosclerosis, arterial hypertension, coronary ischemia, and stroke [4, 5].

According to the number of apnea/hypopnea events (AHI) during one hour of sleep, OSA can be divided into mild (5 ≤ AHI < 15), moderate (15 ≤ AHI < 30), and severe (AHI ≥ 30) [6, 7].

Even if the CPAP (continuous positive air pressure) therapy is the gold standard, the American Academy of Sleep Medicine recommends oral appliance (OA) therapy for patients with mild to moderate OSA and for those with more severe OSA who cannot tolerate CPAP and refuse surgery [8].

OAs have similar treatment efficacy for mild to moderate OSA as CPAP and provided evidence that supports the use of OAs in clinical practice [9].

Mandibular advancement devices (MADs) proved successful in improving AHI and comparison with inactive appliances suggests that mandibular advancement is crucial in terms of establishing efficacy [10–12].

The evidence shows that there is no one MAD design that most effectively improves polysomnographic indices, but that efficacy depends on a number of factors including severity of OSAS, materials and method of fabrication, type of MAD (monobloc/twin blocks), and the degree of protrusion (sagittal and vertical) [10, 13].

As regards the safety, side effects of MAD are reported for time periods up to 5 years [14].

Several studies in literature confirm the short term (≤12 months) therapeutic efficacy of oral appliances (OAs) [15, 16] while there are contrasting results concerning the long term (>12 months) [17–21].

The aim of the present study is to evaluate the long term (48 months) therapeutic efficacy of a soft monobloc mandibular advancement device (sMMAD) in adult patients with mild or moderate obstructive sleep apnea.

## 2. Materials and Methods

At the beginning, a sample of 35 consecutive OSA subjects was selected from adult patients of the Department of Orthodontics of University of Rome Tor Vergata in Rome. Only 28 OSA subjects (6 female and 22 male, mean age 52.2 ± 6.8 years) were selected for this study on the basis of the following inclusionary criteria:

- treatment with the sMMAD for at least 48 months;
- patients affected by a mild to moderate OSA (AHI < 30) and patients affected by a severe OSA (AHI ≥ 30) intolerant to CPAP;
- presence in each arch of at least 10 teeth, for ensuring a proper retention for the oral device;
- no removable dentures;
- no parodontal and temporomandibular diseases;
- body mass index (BMI) < 40;
- no reversible morphological upper airway abnormalities (e.g., enlarged tonsils) as assessed by the ear, nose, and throat (ENT) specialists;
- no medication that could influence respiration or sleep.

Patients were informed about the sMMAD treatment and study protocol and they signed an informed consent. The subjects' rights have been protected.

Diagnosis of OSAS was evaluated by anamnesis and polysomnography (PSG), while daytime somnolence was recorded by the* Epworth Sleepiness Scale (ESS) Questionnaire* (Appendix A). Patients were treated by one author to minimize any method error.

### 2.1. Polysomnographic Analysis

Prior to treatment (T0), a diagnostic polysomnography done using a portable monitoring system was conducted on all selected patients in the Department of Neurophysiopathology of The University of Rome Tor Vergata in Rome by one specialist for monitoring apnea/hypopnea index (total number of apneas-hypopnea events/hour of sleep, AHI).

Clinical (anamnesis and questionnaire ESS) and instrumental (polysomnography) examinations were recorded and used to obtain the BMI (body mass index), ESS, and AHI values in different times: before the beginning of treatment (T0), after 6 months (T1), and 48 months (T2) from the beginning of treatment.

### 2.2. Control Questionnaire

A standardized self-administered questionnaire was addressed to all MAD-fitted OSAS patients. Questionnaire (Appendix B) based on SF-36 quality of life questionnaire and outcome questionnaire [22] was given to obtain information about

- compliance,
- regular use of device (use >8 hours during the night for 5 days in a week was considered regular),
- persistence of OSA symptoms (apneas, snoring, and daily sleepiness),
- improvement or deterioration of the lifestyle due to the sMMAD,
- sMMAD side effects (TMJ pain, excessive salivation, and muscle pain),
- opinions of the patient about the orthodontic therapy.

### 2.3. Technical Features of the Device

**Figure 1:**
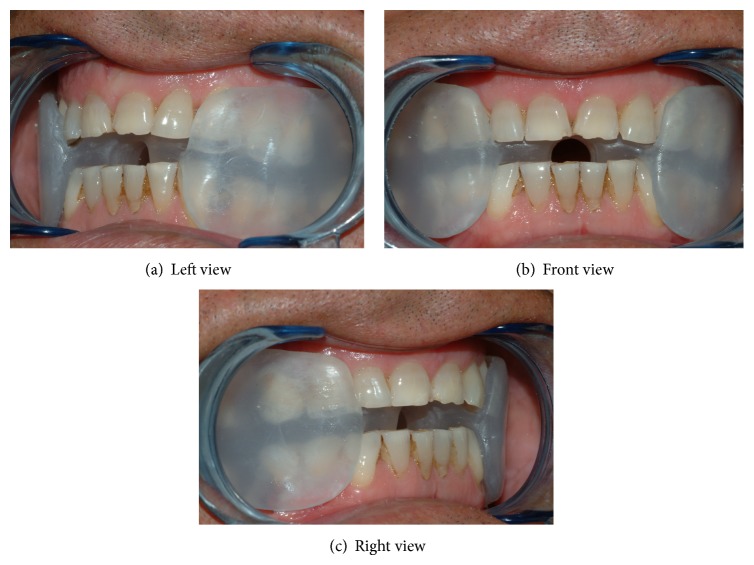
Soft monobloc mandibular advancement device (sMMAD).

The sMMAD used in the current study is a one-piece device made with thermoplastic material (Plastulene). This device covers the tongue and buccal and occlusal surfaces of the posterior teeth while buccal surface of anterior teeth is not covered by resin. In correspondence of the central incisors area, the device has a hole that allows the passing of the air. This MAD is not adjustable device (Figures 1(a), 1(b), and 1(c)).

sMMAD is realized following the anatomic variability of each patient and not referring to the standard parameters related to the degree of the advancement and the vertical dimension.

Alginate impressions, a centrical wax bite, and a protrusive wax bite were taken by only one author for the construction of each appliance. The protrusion and the opening of the bite were individually adjusted for each patient according to a construction bite; in the sagittal plane, sMMAD were designed to hold the mandible to 75% of maximal protrusion and in the vertical plane about 6 mm, to ensure retention of the device.

The George Gauge, an instrument for bite registration, was used to construct an index of the anterior and vertical positions of the mandible [23].

Each device was fitted and then reviewed 4–6 weeks later. Patients were instructed to use the sMMAD every night during sleep [24].

### 2.4. Statistical Analysis

Data analysis was performed by using the Statistical Package for Social Sciences version 22.0 software (SPSS Inc., Chicago, III).

Descriptive statistics of all selected variables (EBM, ESS, and AHI) were computed for T0 (prior to treatment), T1 (after 6 months), and T2 (after 48 months) periods. Shapiro-Wilks normality test showed a normal distribution of the data, expressed as the mean (*M*) ± standard deviation (SD), and thus parametric tests were used in statistical evaluation.

Level of significance was *P* ≤ 0.05 (Table 1).

## 3. Results

The apnea/hypopnea index (AHI) mean was 12,3 ± 3,6 before treatment (T0); 9,4 ± 3,5 after 6 months (T1); and 10,4 ± 2,1 after 48 months (T2).

Body mass index mean (BMI) was 25,7 ± 1,6 at T0; 26,3 ± 2,1 at T1; and 25,4 ± 1,4 at T2.

Epworth Sleepiness Scale index mean (ESS) was 7,4 ± 2,9 before treatment (T0); 4,6 ± 1,9 after 6 months (T1); and 6,5 ± 2,9 after 48 months (T2).

The statistical analysis showed a significant decrease in BMI value between T1 and T2 (*ρ* = 0,012), an increase of ESS value between T1 and T2 (*ρ* = 0,012), and a significant improvement and decrease of AHI between T0 and T1 (*ρ* = 0,010) and between T0 and T2 (*ρ* = 0,013) (Table 1).

By polysomnography evaluation, 10% of patients were stable (AHI T0 = AHI T2), 70% improved (AHI T2 < AHI T0), and 20% worsened (AHI T2 > AHI T0).

Results concerning the Control Questionnaires (Appendix B) showed that 40% of patients did not wear assiduously the sMMAD while 60% of patients used it constantly; 20% had OSA symptoms (apneas, snoring, and daily sleepiness) versus 80% that had an improvement of their own lifestyle with the sMMAD treatment.

About side effects (TMJ pain, excessive salivation, and muscle pain) in long term, only the 20% of patients found it uncomfortable in the early stages versus 80% that found it comfortable.

## 4. Discussion

OSA represents the most severe syndrome related to obstruction of the upper airway. If left untreated, it can lead to cardiovascular complications (increased heart rate and blood pressure, abnormal heart removal, and high probability of heart stroke), cerebral and mental complications due to insufficient oxygenation, and hormonal complications due to a reduction of thyroid hormones, growth hormone, LH, aldosterone, cortisol, and testosterone. Disruption of normal sleep architecture, caused by hypopneic-apneic events during the night and the resulting daytime sleepiness, causes a poor job performance, an increased risk of traffic accidents, headaches, and neurocognitive deficits [7, 25].

MADs (mandibular advancement devices) are a type of intraoral device conceived as an alternative option to the ventilation therapy (CPAP), pharmacological and surgical treatment of mild to moderate OSA [8].

Mandibular advancement devices (MADs) are little devices of dimensions corresponding to the patient's mouth to apply before sleep, which may prevent the adhesion of tongue to pharyngeal posterior wall and maintain a certain expansion of inferior oropharyngeal zone. They are constructed so that the lower jaw is positioned several millimetres anteriorly, with a variety of materials that included rigid and soft methyl methacrylate material, thermoplastic material that softens in hot water, and silicone. MADs differ with regards to design in one-piece (monobloc) or two-piece (bibloc, a separate appliance for each arch), full or partial occlusal coverage and can be custom-made or pre-fabricated. Retention of the appliance is usually provided by claps or friction grip. The one-piece appliance design MAD fixes the mandible rigidly in an anterior position (no adjustable MADs). Most two-piece appliances are sagittally adjustable, thereby allowing for individual titration and possibly greater mandibular advancement. As a matter of fact, until now, few researches have clearly demonstrated any great advantage of one design over another [26].

Several studies in literature confirm the short term (≤12 months) therapeutic efficacy of oral appliances (OAs) [15, 16] while there are contrasting results concerning the long term (>12 months) [17–21].

Even if 80% of patients declare having an improvement of lifestyle after sMMAD treatment at T2, our data, about AHI values during the time, show a low clinical significance. The course of the disease is slowed down by the sMMAD in the first year of treatment and remains stable in the following years.

According to our findings, there are some authors that observed a tendency for the efficacy to reduce over time [13, 18].

Aarab et al. confirm the efficacy of MAD therapy after 1 year of treatment comparing two groups of patients: a group of 21 subjects in MAD therapy and a group of 22 subjects in nCPAP therapy. Results of this study show improvements in AHI after 1 year of therapy in each group that remained stable over time (*P* = 0.650) [16].

Fransson et al. (2003) evaluate subjective discomfort and somnographic measures of 65 patients with obstructive sleep apnea and snoring problems who had been treated for 2 years with an adjustable one-piece device (monobloc) of heat-cured methyl methacrylate mandibular advancement device (MPD). They conclude that MAD treatment of OSAS sufferers is associated with a significant reduction in subjective complaints like disturbingsnoring, apneas, and daytime tiredness, with an improvement in the quality of night sleep, and a significant reduction in oxygen desaturation index (ODI). In addition, favorable 6-month results were unchanged after 2 years [19].

In a recent study Brette et al. evaluate a long term assessment of a two-piece adjustable device called OPM4 J, measuring symptoms, compliance rate, and adverse effects in a cohort of 140 consecutive patients treated with the device for an average period of three years. Out of 140 patients aged 62 ± 10 years with body mass index (BMI) 27 ± 4 kg/m^2^ and initial apnea/hypopnea index (AHI) 27 ± 16, complete reversal of OSAS was achieved in 65% [20].

Rose et al. in their study investigate the long term efficacy of a monobloc acrylic oral appliance, the Karwetzky activator, on respiratory and sleep parameters in 26 patients with obstructive sleep apnea (OSA). Results of their study show that the initial improvement (6–12 weeks) of respiratory parameters statistically decreased after 6 to 12 months and 18 to 24 months, respectively, and a reduced efficiency was also noted in the subjective assessment. Authors suggest that, for an efficient therapy, nocturnal control PSG studies at regular intervals are essential to assure continued therapeutic efficacy. Moreover, it is important to find the several factors that could be responsible for the long term lessening of efficacy. OSA usually worsens with increased age, body mass index, and alcohol consumption and more time spent sleeping supine [13].

In contrast, Fransson et al. find no significant difference in outcome between non-supine-dependent and supine dependent OSA patients at baseline [19].

After 48 months of treatment, patients of this study received a Control Questionnaire to evaluate the efficacy of sMMAD on lifestyle quality. 80% of patients showed a reduction in daytime sleepiness and snoring, a decrease in discomfort during the night, and an improvement of lifestyle and sleep.

In contrast to our results, other studies demonstrated pain during mandibular function, joint clicks, locking, headache, increased salivation, dryness of lips and mouth and tooth mobility, or occlusal alterations [19, 27].

An unfavorable element common to the devices used is deterioration that was not detected in the sMMAD after 48 months [13, 27–29].

## 5. Conclusions

This study shows that treatment with the sMMAD is a therapeutic solution with stable effects and statistically significant improvements in the long term (48 months) for patients suffering from mild or moderate obstructive sleep apnea (OSA). Anyway the clinical significance of the improvements is still low in some subjects.

The sMMAD therapy success in the long term could be due to following reasons:

- accurate selection of OSA patients;
- appropriate therapeutic protocol;
- sMMAD technical features (individual, soft monobloc, comfortable, not adjustable).

The long term prognosis of OSA in adult patients depends on compliance of patient, concomitant presence of other systemic diseases, position taken during sleep, weight gain, status of periodontal tissues, and amount of restorations.

It is important to carry out an annual check to detect possible alterations caused by the MAD and predict potential changes in polysomnographic variables.

## Figures and Tables

**Table 1:** Distribution of means (M) and standard deviation (SD) before treatment (T0), after 6 months (T1), and 48 months (T2) of treatment.

	T0		T1		T2		Statistical analysis		
	Mean	SD	Mean	SD	Mean	SD	T0-T1	T0–T2	T1-T2
BMI (body mass index)	25,8	1,7	26,3	2,2	25,5	1,4	NS	NS	∗

ESS (Epworth Sleepiness Scale)	7,5	2,9	4,6	1,9	6,5	2,9	∗∗	NS	∗

AHI (apnea/ hypopnea index)	12,4	3,6	9,5	3,6	10,4	2,1	∗	∗	NS

^*^0,05.

^**^0,01.

^***^0,001.

NS = not significant.

## Appendices

### A. Epworth Sleepiness Scale

The Epworth Sleepiness Scale is a prescreening tool used to determine the level of daytime sleepiness. A score of 10 or more is considered sleepy.

Use the following scale to choose the most appropriate number for each situation:

0 = would never doze or sleep,
1 = slight change of dozing or sleeping,
2 = moderate chance of dozing or sleeping,
3 = high chance of dozing or sleeping.

Fill in your answers and see where you stand.

*Situation: Chance of Dozing or Sleeping*

- Sitting and reading.
- Watching TV.
- Being a passenger in a motor vehicle for an hour or more.
- Lying down in the afternoon.
- Sitting and talking to someone.
- Sitting quietly after lunch (no alcohol).
- Stopping for a few minutes in traffic while driving.

*Total Score (Add the Scores Up)*

This is your Epworth score.

### B. Control Questionnaire

Name and Surname … Age …

1. How many times do you use your OA (oral appliance) for OSAS? …
2. How many hours a day do you use it? …
3. Do you use it constantly? …
4. If no, Why? …
5. Do you think that your lifestyle is better using this OA? SI NO
6. Are still there any symptoms of OSAS? SI NO
7. If yes, What? …
8. What kind of effects did you have during your treatment?
  - Discomfort.
  - Temporomandibular disorders.
  - Muscle pain.
  - Excessive salivation.
  - Tooth mobility.
  - Altered occlusion.
  - Difficulty falling asleep.
  - Extrusion posterior teeth.

## Abbreviations

sMMAD: Soft monobloc mandibular advancement device
OSA: Obstructive sleep apnea
BMI: Body mass index
ESS: Epworth Sleepiness Scale
AHI: Apnea/hypopnea index
OA: Oral appliance
CPAP: Continuous positive air pressure.
